# Impact of a Daily SMS Medication Reminder System on Tuberculosis Treatment Outcomes: A Randomized Controlled Trial

**DOI:** 10.1371/journal.pone.0162944

**Published:** 2016-11-01

**Authors:** Shama Mohammed, Rachel Glennerster, Aamir J. Khan

**Affiliations:** 1 Interactive Research and Development, Karachi, Pakistan; 2 Abdul Latif Jameel Poverty Action Lab, Massachusetts Institute of Technology, Cambridge, MA, United States of America; Chinese Academy of Medical Sciences and Peking Union Medical College, CHINA

## Abstract

**Importance:**

The rapid uptake of mobile phones in low and middle-income countries over the past decade has provided public health programs unprecedented access to patients. While programs have used text messages to improve medication adherence, there have been no high-powered trials evaluating their impact on tuberculosis treatment outcomes.

**Objective:**

To measure the impact of *Zindagi SMS*, a two-way SMS reminder system, on treatment success of people with drug-sensitive tuberculosis.

**Design:**

We conducted a two-arm, parallel design, effectiveness randomized controlled trial in Karachi, Pakistan. Individual participants were randomized to either *Zindagi SMS* or the control group. *Zindagi SMS* sent daily SMS reminders to participants and asked them to respond through SMS or missed (unbilled) calls after taking their medication. Non-respondents were sent up to three reminders a day.

**Setting:**

Public and private sector tuberculosis clinics in Karachi, Pakistan.

**Participants:**

Newly-diagnosed patients with smear or bacteriologically positive pulmonary tuberculosis who were on treatment for less than two weeks; 15 years of age or older; reported having access to a mobile phone; and intended to live in Karachi throughout treatment were eligible to participate. We enrolled 2,207 participants, with 1,110 randomized to *Zindagi SMS* and 1,097 to the control group.

**Main Outcome:**

The primary outcome was clinically recorded treatment success based upon intention-to-treat.

**Results:**

We found no significant difference between the *Zindagi SMS* or control groups for treatment success (719 or 83% vs. 903 or 83%, respectively, p = 0·782). There was no significant program effect on self-reported medication adherence reported during unannounced visits during treatment.

**Conclusion:**

In this large-scale randomized controlled effectiveness trial of SMS medication reminders for tuberculosis treatment, we found no significant impact.

**Trial Registration:**

The trial was registered with ClinicalTrials.gov, NCT01690754.

## Introduction

Tuberculosis is the second-leading cause of death from infectious diseases globally, with nine million people infected and 1.5 million deaths in 2013 [1]. Treatment for drug-sensitive tuberculosis lasts six to eight months and can result in difficult side effects. Failure to adhere to treatment can result in continued transmission, the development of multidrug-resistant tuberculosis, or death. The World Health Organization recommends directly observed therapy to promote adherence, where a pre-assigned treatment supporter watches each patient take his/her daily medication. However, evidence for the effectiveness of this method is inconclusive [2].

The rapid uptake of mobile phones in low- and middle-income countries has provided public-health programs unprecedented access to patients. Mobile phone-based interventions to improve medication adherence have been adopted for many diseases, with mixed results[3–6]. Most trials are inadequately designed, insufficiently powered, or are restricted to high-income countries [3–6]. The most rigorous trials evaluating mobile phone-use for treatment compliance in developing countries exist for adherence to antiretroviral therapy (ART) for people living with Human Immunodeficiency Virus (HIV). Meta-analysis that combines two positive trials in Kenya [7,8] and one null result from Cameroon [9] found an overall positive impact of weekly Short Message Service (SMS, or text message) reminders [10–12]. A more recent study of interactive automated voice reminders and pictorial messages in India, found no impact on adherence to ART [13].

While there is considerable interest in the potential of mobile phone-based interventions to improve tuberculosis treatment adherence, rigorous evidence on their impact is limited [14–17]. Recently, a limited cluster randomized trial in China found that, while medication monitor reminders led to improved drug compliance for tuberculosis patients, daily two-way SMS reminders did not [18]. Our effectiveness trial gauged the impact of a two-way interactive SMS medication reminder system *(Zindagi SMS)* on the treatment success of people with drug-sensitive tuberculosis.

## Methods

### Study Design and Participants

Participants were recruited into the randomized controlled trial through a large tertiary center (the Indus Hospital), nine public facilities, and a network of private General Practitioner (GP) clinics and private laboratories in Karachi.

To be eligible, participants had to be newly diagnosed with smear or bacteriologically positive pulmonary tuberculosis; 15 years of age or older; report having access to a mobile phone; and intend to live in Karachi throughout treatment. To allow *Zindagi SMS* to help establish habits early, participants had to be on treatment for less than two weeks. To minimize spillovers, patients with another household member in the study were ineligible. Enrolment continued until the predetermined sample size was met.

Eligible participants were consented using an oral consent form, a copy of which was given to the participant. Oral consent was solicited instead of written consent, as our sample was a low-literate population. The trial and oral consent procedure was approved by the research ethics boards at Interactive Research and Development (IRD) in Karachi, Pakistan and the Massachusetts Institute of Technology (MIT) in Cambridge, USA. The trial was registered with ClinicalTrials.gov, NCT01690754.

### Randomization and Masking

Once a patient consented to participate, a study representative entered identifying information on a mobile phone-based enrolment form. Individual participants were randomized to either the *Zindagi SMS* or control groups, using predetermined list on the study server that was generated using simple randomization. The research team was blinded to the allocation sequence generated. If mobile data connectivity was interrupted (10% of enrolments), the study representative called their supervisor, who entered the identifying information into Microsoft Excel and generated the group assignment using the randomization function. In both cases field staff who interacted directly with patients had no ability to influence the randomized allocation. The randomization status of individual participants was not shared with treating clinics by the research team.

### Procedures

All study participants received the standard of care provided by their clinic. Initially, participants received NTP’s recommended eight-month treatment regimen. However, NTP guidelines changed in the second quarter of 2012 to a six-month regimen.

*Zindagi SMS* used two-way reminders to encourage patients to actively engage with reminders, rather than passively read and potentially ignore them. It enabled the study team to identify non-responsive patients for phone calls to encourage participation. *Zindagi SMS* sent enrolled patients daily reminders at a time of their choosing. Messages were in Urdu using English script and included a daily motivational message followed by a reminder to respond via SMS or, after September 2011, a missed (unbilled) call to indicate they had taken their medication. Fourteen messages were randomized and sent to participants. Based on feedback from our pilot, tuberculosis was not mentioned in the messages due to stigma [19]. For example, one message said, “*Your health is in your hands*. *Take your medication and remember to respond by SMS or a missed call*.*”* SMS responses were not verified for content. Participants were offered PKR 60 (USD 0.60) per month to cover the costs of responding. Initially, the participants were asked to pick up reimbursements at their clinic, but from October 2013, reimbursements were transferred directly to participants’ phones.

Once *Zindagi SMS* received a response or a missed call, a confirmatory SMS message was sent to the respondent. If the patient did not respond within two hours, a second reminder was sent. A third and final reminder for the day was sent after two additional hours of non-responsiveness. Members of our study team phoned participants who did not respond for seven days.

Participants were interviewed at their household for a more extensive baseline. Ninety percent of completed baseline surveys were conducted within 30 days of enrolment. Once a month, study enumerators attempted surprise visits to participants’ households to conduct a midline survey. Sputum samples, independent from those collected by the treating clinics, were collected for patients at various points in their treatment. Given the difficulties in finding participants at home and that they could not always produce sputum, there is considerable variation in the sputum samples available per patient. An endline survey was conducted after the completion of participants’ treatment period. Participants who were reported as having defaulted or transferred out from treatment were surveyed again between September and November 2014 to record whether they continued their treatment.

The enrolment form was entered on mobile phones, with paper forms as backup. Other surveys were collected on paper and double-entered into a Microsoft Access database.

### Outcomes

The primary outcome was programmatically defined treatment success as recorded in clinic registers provided by the NTP, the global standard for assessing tuberculosis programs. Treatment success is defined as the sum of patients clinically reported as cured (i.e. patient whose sputum smear or culture was positive at the beginning of treatment but who was smear- or culture-negative in the last month of treatment and on at least one previous occasion) or treatment completed (i.e. a patient who completed treatment but who does not have a negative sputum smear or culture result in the last month or treatment and on at least one previous occasion). In our secondary analysis, we explored the full range of clinically recorded treatment outcomes (cured, treatment completed, default, died, treatment failure and transferred out). In particular, *Zindagi SMS* might have increased success by reducing default (i.e. a patient whose treatment was interrupted for two consecutive months or more). The limitation of this outcome is that because registers of NTP-reporting clinics in Pakistan are not linked, patients who are reported as having default or transferred out could have continued treatment elsewhere. As a robustness check we therefore explored *adjusted* treatment outcomes for treatment success and default, substituting self-reported for clinically recorded outcomes for the participants interviewed.

To explore potential mechanisms of impact (or lack of impact), we also gauged adherence as a secondary outcome by asking participants whether they had taken their medication in the last 24 hours during home visits. While self-reported adherence can be unreliable, there is no reason to believe misreporting is systematically different among those assigned to *Zindagi SMS* or the control group. During survey visits between February and April 2012, we conducted IsoScreen tests, which detect isoniazid metabolites in urine samples to gauge whether tuberculosis drugs were taken within the past 24 hours. Isoniazid is always included in first-line tuberculosis treatment. We compared IsoScreen results with self-reported adherence on the same visit. While IsoScreen tests were conducted on a non-random sample, they give us an indication of the reliability of self-reported adherence.

Finally, we collected self-reported psychological and physical health measures as secondary outcomes. We used a four-point Likert scale for patients’ difficulty in completing a range of physical tasks and how supported they felt during treatment, a picture of a five-rung ladder with six compartments between rungs for likelihood of being cured, and images of five faces for how healthy they felt. Questions on participants’ ability to complete tasks and self-reported health were asked at baseline, midline, and endline; the likelihood of being cured asked at baseline and midline; and how supported participants felt asked at endline.

### Statistical Analysis

We calculated a minimum sample size of 1,094 participants in each study arm, using power of 80%, minimum detectable effect size (MDE) of five percentage points, and treatment success rate in the control group of 75%. An MDE of 5 percentage points was chosen because a smaller impact was considered unlikely to motivate policy change. Data were analyzed using intention-to-treat, i.e. allocation to treatment not actual take up. We used the χ^2^ test for differences in proportions for the analysis of the primary outcome. In comparing sputum samples, we ran ordinary least squares regressions, after controlling for days in the study and regimen type.

We also assessed whether *Zindagi SMS* had differential impacts on treatment success by gender, indicators of quality of care, and mobile phone access. In analyzing our secondary outcomes, we ran ordinary least squares regressions on each outcome to test for program effects, after controlling for days since enrollment and regimen type. To adjust for multiple hypotheses testing we used the Bonferroni correction and the less conservative Westfall and Young free step-down resampling method [20].

Statistical analysis was conducting using STATA/IC version 12.0. A p-value of <0.05 was considered statistically significant.

## Results

**Fig 1:**
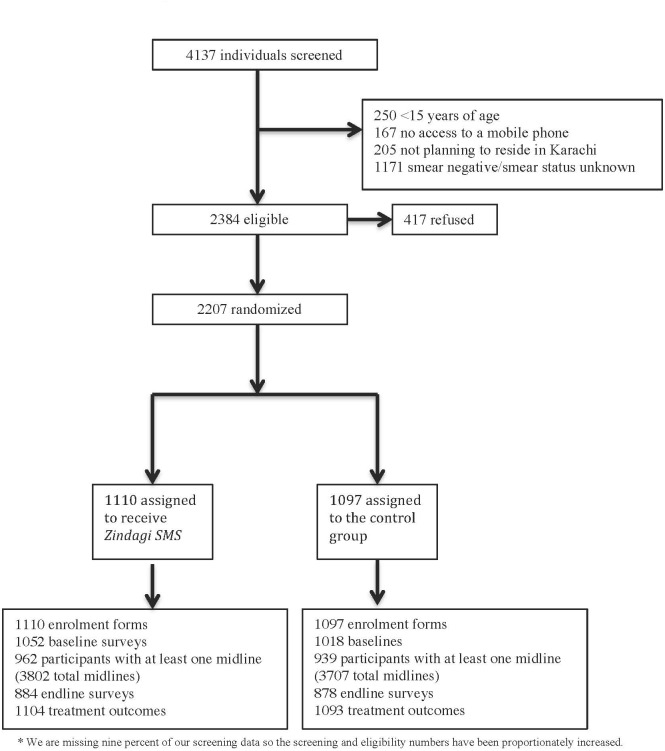
Trial Profile.

We enrolled 2,207 participants into the study between March 18, 2011 and February 25, 2014 (Fig 1). Both groups had similar baseline characteristics (Table 1).

**Table 1:** Demographic characteristics of participants enrolled in the trial (2011–2014, Karachi, Pakistan).*

		SMS Group (n = 1110)	Control Group (n = 1097)
		n (%)	n(%)
Female		561 (51%)	518 (47%)
Age (mean/SD) ^†^		33 (16)	33 (16)
Urdu is mother tongue		529 (48%)	549 (50%)
Clinic type			
	Indus Hospital	404 (36%)	385 (35%)
	GP clinic/private^‡^ laboratory	190 (17%)	193 (18%)
	Public tuberculosis clinic	516 (46%)	519 (47%)
6 Month treatment regimen		764 (69%)	777 (71%)
Assigned a treatment supporter		102 (10%)	106 (10%)
Own mobile phone		540 (49%)	565 (52%)
Schooling			
	No school	517 (49%)	475 (47%)
	Primary (class 1–5)	108 (10%)	115 (11%)
	Secondary (class 6–10)	325 (31%)	307 (30%)
	Tertiary (above class 10)	77 (7%)	101 (10%)
	Religious school	15 (1%)	16 (2%)

*There are 138 missing values for assigned a treatment supporter, 145 missing values for age; 146 missing values for no school and religious school; and 151 missing values for primary, secondary, and tertiary.

^†^SD = standard deviation

^‡^GP = private general practitioner

**Fig 2:**
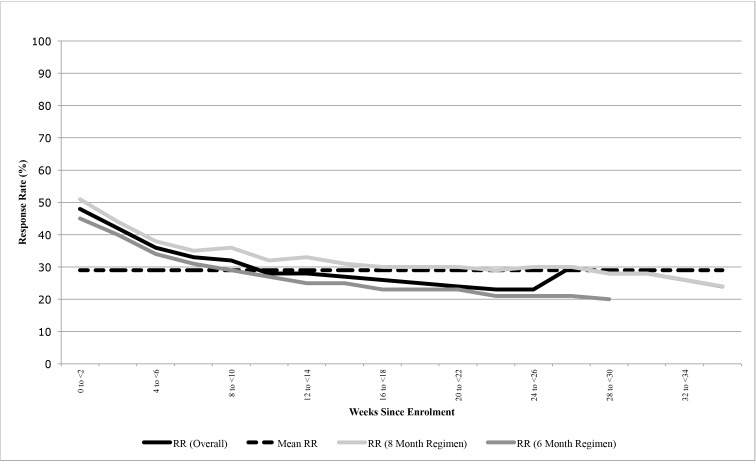
Response rates over time in treatment.

As an effectiveness trial we sought to replicate implementation conditions, as it would exist at scale. The *Zindagi SMS* system sent reminders or received responses for 174,284 patient days to participants during their estimated treatment duration (180 days for the six-month regimen or 240 days for the eight-month regimen). With perfect implementation, reminders should have been sent for a total of 220,560 patient days, suggesting the system was successfully implemented for 79% of patient days. Missed reminders were due to system failures, administrative shortfalls, or GPRS outages in the city mobile (14%); participants asking to leave the system or dying (3%); participants opting out of receiving reminders at enrolment (2%); and participants not knowing their phone number at enrolment and failing to share their number subsequently (2%). Of the 1,069 participants who were sent messages, 912 (85%) responded at least once. Of the participants that were on the system throughout their treatment, the mean response rate (calculated as the number of responses received over the number of reminders sent per patient) was 29%, ranging from 0 to 99%. Over the course of treatment, average response rates fell from 48% in the first two weeks to 24% (eight-month regimen) and 20% (six-month regimen) in the last two weeks (See Fig 2).

**Fig 3:**
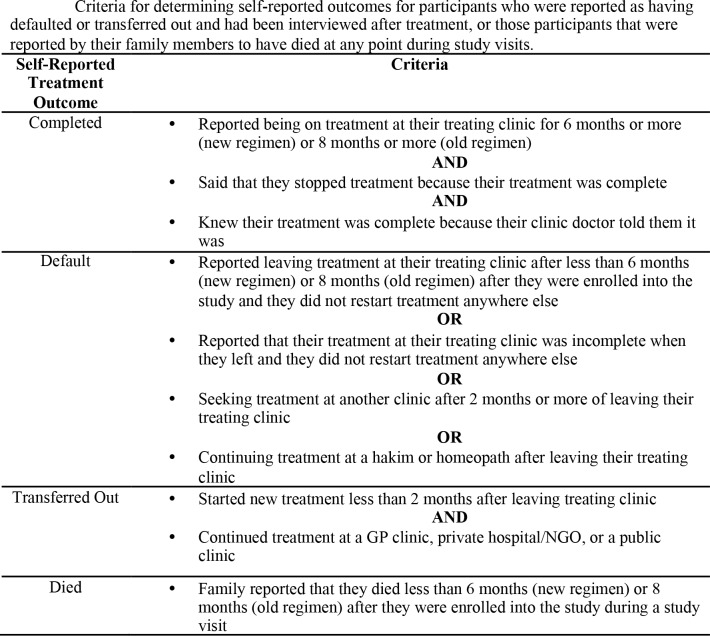
Criteria for determining self-reported outcomes.

There were no significant differences in treatment outcomes between participants in the *Zindagi SMS* group and the control group (Table 2). As a robustness check, we substituted the clinically recorded outcomes with self-reported outcomes for participants who defaulted or transferred out of treatment that we were able to interview, or whose family members reported the participant had died. Of the 283 participants that were reported as having defaulted or transferred out, we interviewed 130 (46%); 49 participants (17%) had died; 22 (8%) refused to be interviewed; and we were unable to locate 82 (29%). Self-reported outcomes were categorized using the criteria in Fig 3. When we adjusted treatment outcomes to reflect the self-reported outcomes, the default rate reduced in both groups but there were still no significant differences in outcomes between the two groups (Table 2). We also examined independent sputum samples between the groups. We collected 1191 sputum samples for participants (603 in the *Zindagi SMS* group and 588 in the control group). Of these, one hundred and ninety samples were excluded because they were primarily saliva, rather than sputum (104 in the *Zindagi SMS* group and 86 in the control group). There was no statistically significant difference between sputum results for both groups (p = 0.762).

**Table 2:** Clinically-recorded treatment success between *Zindagi SMS* and control groups (2011–2014, Karachi, Pakistan).

	Clinically recorded treatment success					Clinically recorded treatment success adjusted for self-reported outcomes				
	*Zindagi SMS*		Control Group			*Zindagi SMS*		Control Group		
	n	*%*	N	%	p-value	n	%	n	%	p-value
Treatment success	917	83%	903	83%	0.782	923	84%	911	83%	0.871
Treatment complete	332	30%	325	30%	0.863	339	31%	333	30%	0.903
Cured	585	53%	578	53%	0.960	584	53%	578	53%	0.994
Default	108	10%	103	9%	0.775	74	7%	80	7%	0.572
Died	19	2%	19	2%	0.975	38	3%	29	3%	0.282
Treatment Failure	27	2%	29	3%	0.758	26	2%	29	3%	0.655
Transfer Out	33	3%	39	4%	0.446	43	4%	44	4%	0.875
Total	1104		1093			1104		1093		

There was no significant program effect in subgroups for gender, any of the indicators relating to quality of care, or access to a mobile phone after adjusting for multiple subgroups using the Bonferroni correction (adjusted p-value of 0.003 for 17 subgroups) or the Westfall and Young free step-down resampling correction (Table 3). We also created a single index of quality of care from relevant variables and another for mobile phone access to test for subgroup effect but again found no significant treatment effects for subgroups. (S1 Appendix)

**Table 3:** Sub-group analysis using treatment success as the outcome (2011–2014, Karachi, Pakistan).

		*Zindagi SMS* Group		Control Group		*Zindagi SMS*	naive	FWER^‡^^-^ adjusted
Sub-group		N	*%*	n	*%*	coefficient	p-value*	p-value
*Sex*								
	Male	438	80%	468	81%	-0.012	0.618	0.999
	Female	479	86%	435	84%	0.019	0.394	0.974
*Quality of care*								
	Indus Hospital	317	79%	301	78%	0.005	0.872	1
	GP Clinic/Private Lab	180	95%	167	87%	0.082	0.006^†^	0.069
	Public TB Clinic	420	82%	435	84%	-0.023	0.331	0.954
	Assigned a treatment supporter	89	87%	84	79%	0.080	0.124	0.698
	Not assigned a treatment supporter	793	84%	768	84%	-0.004	0.815	1
	Reminded to take medication (with one month after enrolment)	355	82%	324	83%	-0.011	0.689	1
	Not reminded to take medication (within one month after enrolment)	436	87%	446	84%	0.022	0.318	0.953
*Access to a mobile phone*								
	Own mobile phone	450	84%	474	84%	-0.005	0.806	1
	Don’t own mobile phone	467	82%	429	81%	0.016	0.502	0.994
	No schooling	416	81%	381	80%	0.004	0.875	1
	Any schooling	461	88%	469	87%	0.006	0.758	1
	At least one literate person in the household	753	85%	744	84%	0.008	0.629	0.999
	No literate people in the household	129	80%	108	81%	-0.016	0.736	1
	Can send SMS (within month of enrolment)	263	88%	232	87%	0.014	0.624	0.999
	Cannot send SMS (within first month of enrolment)	528	83%	538	82%	0.001	0.958	1

*Bonferroni correction p-value (with 17 subgroups): 0.003

^†^p<0.05

^‡^Family-wise error rate^20^

We compared the results of 159 IsoScreen tests with self-reported adherence during the same study visit. IsoScreen tests indicated that 17% of those who said they had taken their drugs in the past 24 hours had not, indicating over-reporting. However, these results were not statistically different by treatment arm.

There were no significant program effects in self-reported medication adherence or any of our other secondary outcomes, after adjusting for multiple hypotheses using the Bonferroni correction (p-value of 0.001 for five hypotheses) or the Westfall and Young correction (Table 4).

**Table 4:** Secondary outcomes between the *Zindagi SMS* and control groups (2011–2014, Karachi Pakistan).

	Took medication in the last 24 hours^†^	Perceptions on likelihood of being cured^†^ (6 = very likely, 1 = not likely)	How healthy they felt^†^ (5 = very healthy, 1 = very unhealthy)	Ease of completing tasks^†^ (4 = no difficulty, 1 = lot of difficulty)	How much support was received^†^ (4 = lot of support, 1 = no support)
Zindagi	0.002	-0.008	-0.012	-0.017	0.020
Naïve p-value*	0.772	0.473	0.423	0.036^‡^	0.521
FWER^§^ adjusted p-value	0.89	0.89	0.89	0.162	0.89
N (surveys)	11,301	9,560	11,324	11,235	1658
N (patients)	2091	2068	2091	2088	1658

*Bonferroni correction p-value (with 5 hypotheses): 0.001

^†^ Controlling for the length of the regimen, days in the study, and days in the study-squared.

^‡^p<0.05

^§^Family-wise error rate [20]

## Discussion

We found no significant impact of SMS medication reminders on treatment success, other treatment outcomes, self-reported adherence, or self-reported physical and psychological health. There were no statistically significant impacts of reminders on treatment success within a variety of subgroups after adjusting for multiple hypothesis testing.

With an attrition rate of less than 1%, similar across arms, on our primary outcome variable, the results are very robust. The inclusion of participants from public clinics, private GP clinics, laboratories, and a large philanthropic hospital contributes to its external validity.

A limitation of our trial was the lack of an objective adherence measure. Adherence was self-reported, which, IsoScreen tests indicated, is an overestimate of actual adherence. However, even if participation in *Zindagi SMS* generated increased adherence, not reflected in self-reports, it did not translate into improved treatment outcomes. In addition, IsoScreen tests (on a nonrandom sample) indicated that misreporting was similar in the *Zindagi SMS* and control groups.

Another potential limitation is that clinics could incorrectly record treatment outcomes to meet expected success rates encouraged by the NTP. However, since clinics were blind to allocation, there is no reason any misreporting was systematically different between treatment arms. Moreover, in our sub-study on participants reported as having defaulted or transferred out, self-reported outcomes were similar in the intervention and control groups. Another potential limitation is that our trial took place in Pakistan, which reports high treatment success rates of 91% in 2012 [1]. Results may differ in countries reporting lower success rates.

Our results were similar to the findings of Liu and colleagues in China that two-way SMS reminders had no impact on medication adherence for tuberculosis patients, though we are able to rule out much smaller effects of SMS reminders [15]. Meta-analysis of studies on HIV and SMS reminders show positive results on of weekly SMS reminders on adherence and clinical outcomes (Finitsis et al 2014 [10], Mbuagbaw et al 2013 [11], Horvath et al 2012) [12]. These reviews draw mainly on three large high quality studies (Finitsis et al also include many small poor quality studies and Horvath et al only includes Lester and colleagues’ [7] and Pop-Eleches and colleagues’[8] trials) and conclude weekly reminders are more effective than daily reminders. The three key studies are Mbuagbaw and colleagues, who test weekly SMS reminders in Cameroon and find no impact [9] Pop-Eleches and colleagues who tested multiple versions of SMS reminders and only found only short messages sent weekly improve adherence [8], and Lester and colleagues find a positive impact of weekly reminders in Kenya [7]. Importantly, in Lester and colleagues, participants also received off-site clinician follow-up if they reported a problem or did not respond to the SMS [7, 21]. Not included in the reviews is Shet and colleagues who test weekly automated telephone reminders in India and find no significant effect [13].

Our study indicates that SMS reminders have no impact on treatment outcomes for patients with drug-sensitive tuberculosis. Participants could operate the system (85% responded at least once), but this did not translate into changed outcomes. We hypothesize that SMS reminders, intended to combat forgetfulness, did not address the underlying factors that contribute to patients leaving treatment. The steady decline of and the low mean of the response rate (29%) suggest that participants tired of daily reminders. It is possible that longer intervals between messages or greater off-site support for remote patients as in the Lester trial might be effective [7, 21–22]. SMS reminders could be more useful for reminding patients of clinic appointments; other forms of appointment reminders (telephone calls, home visits, letters) have helped tuberculosis patients [23]. Finally, SMS reminders could be coupled with financial incentives to motivate patients to stay on course with treatment, especially given the important externalities to the community associated with a patient complying with their treatment regime. Our current study was initially intended to have an arm with incentives for medication adherence, but the technology to link SMS reminders with adherence had manufacturing challenges. Our results suggest pessimism for SMS medication reminders on their own.

In conclusion, we found no impact of SMS reminders on treatment success in tuberculosis patients in the highest-powered such randomized control trial to date.

## Supporting Information

S1 Appendix Additional Sub-group Analysis Using Subgroup Indices. (DOCX)

S1 File CONSORT 2010 Checklist. (DOC)

S2 File Evaluating the Impact of Zindagi SMS on Treatment Outcomes of Tuberculosis: A Randomized Control Trial Protocol. (DOCX)
