# Supplementary material for: Impact of a Daily SMS Medication Reminder System on Tuberculosis Treatment Outcomes: A Randomized Controlled Trial
Source: PLoS One. 2016 Nov 1;11(11):e0162944. doi: 10.1371/journal.pone.0162944 (PMC5089745; doi:10.1371/journal.pone.0162944)
Supplement: S1 Appendix — (DOCX) [file pone.0162944.s001.docx]

**Supporting Information**

**Additional Sub-group Analysis Using Subgroup Indices**

In accordance with our pre-analysis plan (PAP), we also explored sub-groups for sex, vulnerability, high access to mobile phones, and quality of care. While sex was composed of a single variable, the remaining subgroups were based on an index of variables, as per our PAP. We had proposed an additional index of variable in our PAP for participants who were likely to respond to the *Zindagi SMS* system, but were unable to find an index of variables that was a stronger predictor of response rates.

Vulnerability was composed of dummy variables for whether the participant was male, whether they had received any schooling, and the proportion of an index of 22 assets that they had in their household. Participants were considered vulnerable if they had a score of less than the median score of 1.545 on the vulnerability index. Access to mobile phones was composed of dummy variables for whether participants owned a mobile phone, had at least one literate person in their household, and whether they said they knew how to send or receive SMS messages in a survey within their first month of enrolment. Participants were considered to have high access to mobile phones if they had a score on the mobile phone access index greater than the median score of 2. Finally, quality of care was calculated on an index composed of whether they were assigned a treatment supporter by their treating clinic, whether they had someone remind them to take their medication in a survey within their first month of enrolment, and the type of clinic they were being treated at, based on the treatment success rates for the various clinic types in the control group (with a value of 0 for type of clinic if they were being treated at Indus, 0.5 if they were being treated at a public tuberculosis clinic, and 1 if they were being treated at a private general practitioner (GP) clinic or lab). The clinic values were based on the treatment success rates for various clinic types in the control group. However, having a treatment supporter or having had someone remind them to take their medication during the first month since enrolment were not significantly associated with treatment success in the control group. Participants were considered to have a high quality of care if they had a score on the quality of care index above the median score of 1.

The subgroup analysis was conducted using an ordinary least squares regression with an interaction term between group assignment and the subgroup variable. In all four subgroups, there was no significant difference in the treatment success rate between the *Zindagi SMS* and control groups.

Table 1: Subgroup analysis using subgroup indices with tuberculosis treatment success as the outcome variable among patients randomized to receive two-way SMS reminders or standard of care (2011-2014, Karachi, Pakistan).

|  | Subgroups | | | |
| --- | --- | --- | --- | --- |
|  | Male  Coef  (p-value) | Vulnerable  Coef  (p-value) | High mobile access  Coef  (p-value) | Good quality of care  Coef  (p-value) |
| Zindagi | 0.019  (0.420) | 0.020  (0.402) | 0.003  (0.874) | 0.018  (0.407) |
| Subgroup | -0.029  (0.206) | -0.035  (0.132) | 0.043  (0.160) | 0.003  (0.901) |
| Interaction (subgroup*zindagi) | -0.030  (0.346) | -0.029  (0.379) | 0.021  (0.631) | -.0.028  (0.428) |
| n | 2197 | 2038 | 1858 | 1857 |
