# Supplementary material for: Impact of a Daily SMS Medication Reminder System on Tuberculosis Treatment Outcomes: A Randomized Controlled Trial
Source: PLoS One. 2016 Nov 1;11(11):e0162944. doi: 10.1371/journal.pone.0162944 (PMC5089745; doi:10.1371/journal.pone.0162944)
Supplement: S2 File — (DOCX) [file pone.0162944.s003.docx]

**Evaluating the Impact of *Zindagi SMS* on Treatment Outcomes of Tuberculosis: A Randomized Control Trial Protocol**

**Principal Investigators:** Shama Mohammed, Rachel Glennerster, Aamir J. Khan

**Background and Rationale**

In Karachi, Pakistan, the incidence of TB is believed to be particularly high, with13,332 patients registered under the National TB Control Programme (NTP) in 2007. Increasingly, the problem of non-compliance is leading to the emergence of multi-drug resistant TB (MDRTB), a form of TB that is more expensive and difficult to treat. Of 7,185 new pulmonary cases studied in Karachi in 2006, 12% were reported to have defaulted on treatment. The system currently recommended by the World Health Organization (WHO) for tackling the problem of non-compliance is Directly Observed Therapy Short-Course (DOTS), which involves on-site, in-person monitoring of compliance with medication. DOTS programmes in Karachi use health workers, health clinic staff, or treatment supporters, who are usually family members, as monitors. However, there is no way of verifying whether and to what extent patients are complying with their drug regimen.

Remote reminders free patients from their dependence on unreliable health workers. IRD is conducting a randomized control trial to gauge the impact of the *Zindagi SMS* system on treatment outcomes for patients with drug-susceptible TB.

**The *Zindagi SMS* System**

The *Zindagi SMS* reminder system is a two-way medication reminder system for people with drug-susceptible tuberculosis. Participants are sent daily medication reminder SMS messages and are asked to respond with an SMS to indicate that they have taken their medication for the day. If the system does not receive a response within two hours, a second reminder is sent. If the system does not receive a response for another two hours, a third and final reminder is sent. Participants who are non-responsive for a week are followed up with a phone call.

Participants are offered PKR 60 per month, as compensation for the cost of sending SMS messages.

**Research Methods**

This study is a randomized control trial that will take place in Karachi, Pakistan. Participants will be recruited through TB clinics throughout the city.

*Participant Inclusion/Exclusion Criteria*

The sample for this study will be drawn from newly diagnosed TB patients with sputum or bacteriologically-positive drug-susceptible TB who are at least 15 years of age in Karachi, Pakistan. Study participants will be required to own a cell phone or have access to one. To reduce spillover effects, patients will be excluded from the study if another household member is already participating. Finally, to enable follow up surveys, participants will have to intend to reside in Karachi throughout their treatment period.

*Recruitment, Randomization, and Data Collection*

When a clinic diagnoses a new patient with tuberculosis, the study’s clinic representative will inform the participant about the study. If a patient meets the eligibility criteria, they will be read an oral informed consent form and will be asked to participate in the study. Emphasis will be placed on explaining that they can refuse to take part in the study without there being any effect on the standard treatment provided to them by the clinic. They will also be informed that participation will require several interviews over the course of the patient’s treatment. Recruitment of minors (below the age of 18) will require the patient's asset as well as informed oral consent from the patient's parent/guardian.

If a participant consents to participate in the study, clinic representatives will then complete a brief enrolment form, including the patient’s name, age, sex, clinic location, as well as other contact information. Random assignment in this study will occur on a rolling basis as new patients are diagnosed with tuberculosis. Patients will be randomized through an electronic randomization process at the time of enrolment, to prevent personal bias from contaminating the randomization process. Patients will be randomized into either the control arm or the *Zindagi SMS* arm of the study using programs on the clinic representatives' mobile phones. Participants will be informed of their randomization status but it will not be shared with their treating clinics by the study representatives.

If the patient is selected into *Zindagi SMS,* the clinical representative will explain the program to the patient, provide him/her with an informational brochure, and provide him/her with a number to call for assistance.

Data collection for the study will occur in three stages for participants in both the *Zindagi SMS* and control groups: Baseline, Midline Surveys, and Endline surveys. The three stages will occur over the standard course of treatment for tuberculosis. The Baseline survey will occur during Month 1 of treatment, Midline data collection will occur on a monthly basis during Month 2 through to Month 7 of treatment (or Month 5 of treatment for participants on the six-month regimen), and the Endline survey will occur after the completion of the expected treatment period. Sputum samples will be attempted from patients after their first, second, fifth and sixth or seventh months of treatment. Each stage of data collection will be voluntary, include informed consent, and be conducted in the patient’s home or in private to ensure confidentiality. All survey questions will be extensively field-tested prior to use in the study in order to be sure that all material covered is culturally sensitive and respectful of local beliefs.

Baseline Survey: Survey enumerators will attempt to visit newly diagnosed TB patients within a week of diagnosis. The baseline survey will collect background information on socio-demographic factors as well as other health conditions.

Midline Surveys: Compliance checks will be attempted every month between Month 2 and Month 7 (or Month 5 for those on an six month regimen) after enrolment. During these months, research staff will conduct unannounced visits to ask participants whether they took their medication in the past 24 hours, and a range on questions about their treatment, health, mobile phone usage, and employment. Urinalysis tests will also be used to objectively gauge compliance.

Endline Survey: After the end of the expected treatment period, enumerators will revisit study participants to conduct a final endline survey. The endline survey will collect information on their health, treatment, as well as attitudes toward compliance with drug regimes.

Clinical outcomes form: Using the patient's District TB number, clinically reported treatment outcomes will be retrieved through the clinic registers. This will enable use clinically reported results to compare outcomes across treatment and control groups.

*Sample Size*

We calculated a minimum sample size of 1,094 per arm to detect an effect size of 5%, from a treatment completion rate of 75% to 80%.

**Analysis**

Analysis of this study will be conducted using intention to treat. Participants randomized to the *Zindagi* SMS group will be considered a part of that group for analysis, regardless of whether they used the SMS system or not.

*Primary Outcome*

The primary outcome for this study is clinically-recorded treatment outcomes. Treatment outcomes for TB patients include cure, treatment complete, treatment failure, died, transfer out, or default. These are reported by TB clinics to the National TB Control Program on all their patients on treatment. We will look at treatment success rates of patients in our study, as recorded by their TB clinics, to compare the means for these outcomes between our intervention and control groups.

*Secondary Outcomes*

There are a number of secondary outcomes for this study:

Medication adherence: Medication adherence will be measured through urinalysis tests and self-reports by participants on whether they took their medication in the past 24 hours. This data will be collected during unannounced midline visits.

Phyical fitness and mobility: Physical fitness will be measured through questionnaires conducted with participants during household visits each month that they are on treatment. The physical fitness index will look at the self-reported ability of respondents to complete certain tasks.

Pyschological impacts: In order to cause of the psychological impacts of the system, the investigators will look at participants’ perceptions of the likelihood of being cured, how healthy they feel on a given day, and how supported they feel.

Sputum conversion: Sputum samples will be collected and we will look at the difference in when sputum conversion occurs between the *Zindagi SMS* and control groups.

*Sub-group Analysis*

In order to test the differential effects of the intervention on various sub-groups, we will be conducting the following sub-group analysis.

- Gender : Women in Pakistan often have restrictions on their mobility. Therefore, an SMS reminder system may be more beneficial to them, as they receive reminders in the comfort of their homes. Women are also more likely to be at home, thereby enabling greater access to the owner of the mobile phone (if it is not the patient herself), as they will be home whenever the owner of the mobile phone is at home. Alternatively, women may be less comfortable with technology and therefore there may be less likely to use the system. Moreover, TB stigma is more prevalent in women, especially young women, and so they may benefit from a remote system that sends messages to them at home.
- Vulnerable Populations: We will be looking at the impact of the system on vulnerable populations. In order to look at whether a participant is vulnerable, we will be looking at a variety of variables related to education, gender, and socioeconomic status. Less vulnerable respondents may be more likely to benefit from the intervention, as they may have better health-seeking behaviour and so a reminder system may result in them acting on the reminders. Alternatively, there may be more of an impact on those of a lower socio-economic group, as they may have worse health-seeking behaviour to begin with and, therefore, the system may have a greater impact on them. We will construct a family of indicators that measure socioeconomic status and test for a heterogeneous treatment effect by socioeconomic status.
- Access to a Mobile Phone: We will be looking at the impact of the system based on participants’ relative access to a mobile phones. The extent of access to mobile phones will be determined by ownership of the phone and familiarity with SMS at baseline. We will also look at factors such as literacy of the patient, literacy of their household members, and the language they use to SMS. Increased access to the mobile phone may increase compliance, because the patient will personally be able to see the messages, which could result in improved compliance. Alternatively, patients that have less personal access to the mobile phone may be reminded family members who have greater access, which could increase their compliance, as they will be monitored by the owner of the mobile phone.
- Quality of Care: We will also be looking at the quality of care received by patients as a sub-group to analyze. Quality of care will be determined by the clinic the patient receives treatment from, whether or not they have a treatment supporter, and whether there is anyone who reminds them to take their medication. We hypothesize that the lower the quality of care, the more impact the system will have, as it will enhance the quality of their care through reminders. However, if the quality of care is so poor that there is a lack of availability of the medication, then this may result in a reduced impact of the system, as it cannot over-ride issues of a lack of availability of medication.
- People likely to respond to Interactive Reminders: We will also use those who are more likely to respond to SMS reminders as a sub-group to analyze. It may be that those who respond more get a greater benefit from the intervention. Thus, we will look at various socio-demographic variables such as gender, age, socio-economic status, literacy, education, and education of other family members, to see whether these are have a significant correlation with response rates for people in the Interactive Reminders arm. Once that is determined, we will examine the impact of Interactive Reminders on this sub-group.

**Ethical Considerations**

- The study will be submitted to the Interactive Research and Development-Institutional Review Board (IRD-IRB) and the Committee on the Use of Humans as Experimental Subjects (COUHES) at MIT for review.
- Participation will depend on informed voluntary consent. In working with minors aged 15 or over, parental consent will be attained as well as the verbal consent of the participant. Participants will have the option of withdrawing from the study at any time.
- The confidentiality of participants will be maintained. All data will be collected on secure password-protected servers. Paper forms will be stored in locked offices at Interactive Research and Development or the Indus Hospital Research Center. Names or other identifying information will not be used in study reports or papers.
